# Surface Functionalization of Iron Oxide Nanoparticles with Gallic Acid as Potential Antioxidant and Antimicrobial Agents

**DOI:** 10.3390/nano7100306

**Published:** 2017-10-05

**Authors:** Syed Tawab Shah, Wageeh A. Yehye, Omar Saad, Khanom Simarani, Zaira Zaman Chowdhury, Abeer A. Alhadi, Lina A. Al-Ani

**Affiliations:** 1 Nanotechnology & Catalysis Research Centre (NANOCAT), University of Malaya, Block A, Level 3, Institute of Postgraduate Studies Building, Kuala Lumpur 50603, Malaysia; tawab_shah2003@yahoo.com (S.T.S.); linaalani@ymail.com (L.A.A.-A.); 2 Department of Pharmacy, Faculty of Medicine, University of Malaya, Kuala Lumpur 50603, Malaysia; omar79@siswa.um.edu.my (O.S.); abeer@um.edu.my (A.A.A.); 3 Institute of Biological Sciences, Faculty of Science, University of Malaya, Kuala Lumpur 50603, Malaysia; hanom_ss@um.edu.my

**Keywords:** gallic acid, nanoantioxidant, DPPH, functionalization, antimicrobial

## Abstract

In this research, we report the size-controlled synthesis and surface-functionalization of magnetite with the natural antioxidant gallic acid (GA) as a ligand, using in situ and post-synthesis methods. GA functionalization provided narrow size distribution, with an average particle size of 5 and 8 nm for in situ synthesis of gallic acid functionalized magnetite IONP@GA1 and IONP@GA2, respectively, which are ultra-small particles as compared to unfunctionalized magnetite (IONP) and post functionalized magnetite IONP@GA3 with average size of 10 and 11 nm respectively. All the IONPs@GA samples were found hydrophilic with stable aggregation state. Prior to commencement of experimental lab work, PASS software was used to predict the biological activities of GA and it is found that experimental antioxidant activity using 2,2-diphenyl-1-picrylhydrazyl (DPPH) assay and antimicrobial studies using well diffusion method are in good agreement with the simulated results. Furthermore, the half maximal inhibitory concentration (IC50) values of DPPH antioxidant assay revealed a 2–4 fold decrease as compared to unfunctionalized IONP. In addition to antioxidant activity, all the three IONP@GA proved outstanding antimicrobial activity while testing on different bacterial and fungal strains. The results collectively indicate the successful fabrication of novel antioxidant, antimicrobial IONP@GA composite, which are magnetically separable, efficient, and low cost, with potential applications in polymers, cosmetics, and biomedical and food industries.

## 1. Introduction

The role of antioxidants in maintaining healthy cells status is well-defined, with a very large amount of research and published articles [1,2,3,4,5]. The endogenous antioxidant defense system is usually sufficient in handling free radicals in the body, while in disease developing-threshold circumstances, the critical need for exogenous antioxidants rises [5].

The fast-growing field of nanotechnology has recently presented a remarkable resolution that can even surpass exogenous dietary antioxidant sources [5]. Nanoantioxidants constitute the upcoming antioxidant agents for therapeutic and industrial applications [6]. Their powerful activity is believed to present more effective dominance over various Reactive Oxygen Species (ROS) [6].

Of late, researchers have investigated antioxidant activity of various metal-based nanocomposites, such as gold [7,8], platinum [9,10,11], iron [12,13,14], nickel oxide [15], ceria [16,17], and yttria [2,18], for applications as nanoantioxidants. Magnetic IONPs owe protuberant antioxidant activity against oxidative damage-related diseases [19,20,21,22,23,24,25]. However, there are several factors that strongly affect nanomaterials antioxidant activity for instance chemical composition, surface charge, particle size, and coating of the surface [6,26,27,28,29]. The surface coating could be biocompatible, nontoxic and allow targeted drug delivery [30,31,32,33,34,35].

Gallic acid (GA) is a well-known powerful natural antioxidant constituent of various herbs [36,37]. It has versatile applications in medicine, food and pharmaceutical industries because of its unique physiochemical characteristics, such as non-toxicity, biodegradability, abundant availability, and low cost. GA possesses multi-therapeutic protecting capabilities, as well as antioxidant, anti-inflammatory, anticancer, antitumor, antimicrobial, and antidiabetic properties [38,39,40,41,42]. Surface functionalized nanomaterials have demonstrated that attachment of antioxidants results in increased antioxidant activity and bioavailability [43,44]. Previous studies have reported the successful functionalization of GA on silica nanoparticles surface, which was identified as an efficient nanoantioxidant [44,45]. The bimetallic (Ag-Se) nanoparticles functionalized with quercetin and gallic acid were used as antioxidant, antimicrobial, and antitumor agents [46].

Superparamagnetic iron oxide has numerous applications, such as in MRI [19], drug delivery systems [20,21], hyperthermia [23], immunoassay and tissue repair and detoxification [24]. Magnetic nanoparticles loaded with antioxidant enzymes (such as superoxide dismutase (SOD) or catalase (CAT)) have been used as in drug delivery system through magnetic guiding. Magnetically-responsive antioxidant nanocarriers can provide therapeutic guiding of high concentrations of antioxidants to specific locations with elevated levels of ROS [25]. Catalase-loaded magnetic nanoparticles showed rapid cellular uptake and provided increased resistance to oxidative stress damage induced by hydrogen peroxide [2,25]. Magnetic nanoparticles have been used for targeted enzyme therapy, which might be used in the treatment of cardiovascular diseases [25,47] that are related to oxidative damage [2]. Recent studies revealed that iron oxide nanoparticles exhibited antioxidant properties and their activity increased with the decrease in particle size [12].

Biomedical applications need nano-sized particles with high magnetization value and narrow particle size distribution. Furthermore, these nanoparticles require a surface coating which must be biocompatible, nontoxic, and must also allow targeted drug delivery to specific area. Nanoparticles can be stabilized by various protection strategies which could be organic coating, such as poly(ethyleneglycol) [48], polysaccharide [30], dodecanethiol-polymethacrylic acid [21], and chitosan [31], or a coating with an inorganic coating such as silica [44], metal or non-metal, metal oxide or sulphide [32]. This surface coating protects nanomaterials from agglomeration while at the same time functionlizing it [33,34]. The functionalized magnetic nanoparticles can bind to drugs, proteins, enzymes, antibodies, or nucleotides and can be directed to an organ, tissues, or a tumor using an external magnetic field [24,25,47,49].

In this study, we report on IONP functionalized with GA as diverse water-soluble antioxidants which have favorable therapeutic and industrial applications. The resulted IONPs@GA nano-antioxidant has advanced biocompatibility, hydrophilicity, and further synergistic organic-inorganic hybrid antioxidant properties. We investigated the influence of GA in the size-controlled synthesis process and the surface functionalization of Fe_3_O_4_ nanoparticles by the in situ oxidation-precipitation of ferrous hydroxide method. Prediction of biological activities of GA molecule at IONP surface was performed with the Prediction Activity Spectra of Substances (PASS) training set. Analytical results based on antioxidant and antimicrobial activities confirmed the predictions obtained by the PASS program.

## 2. Results

### 2.1. Fourier-Transform Infrared Spectroscopy (FTIR) Analysis

**Figure 1:**
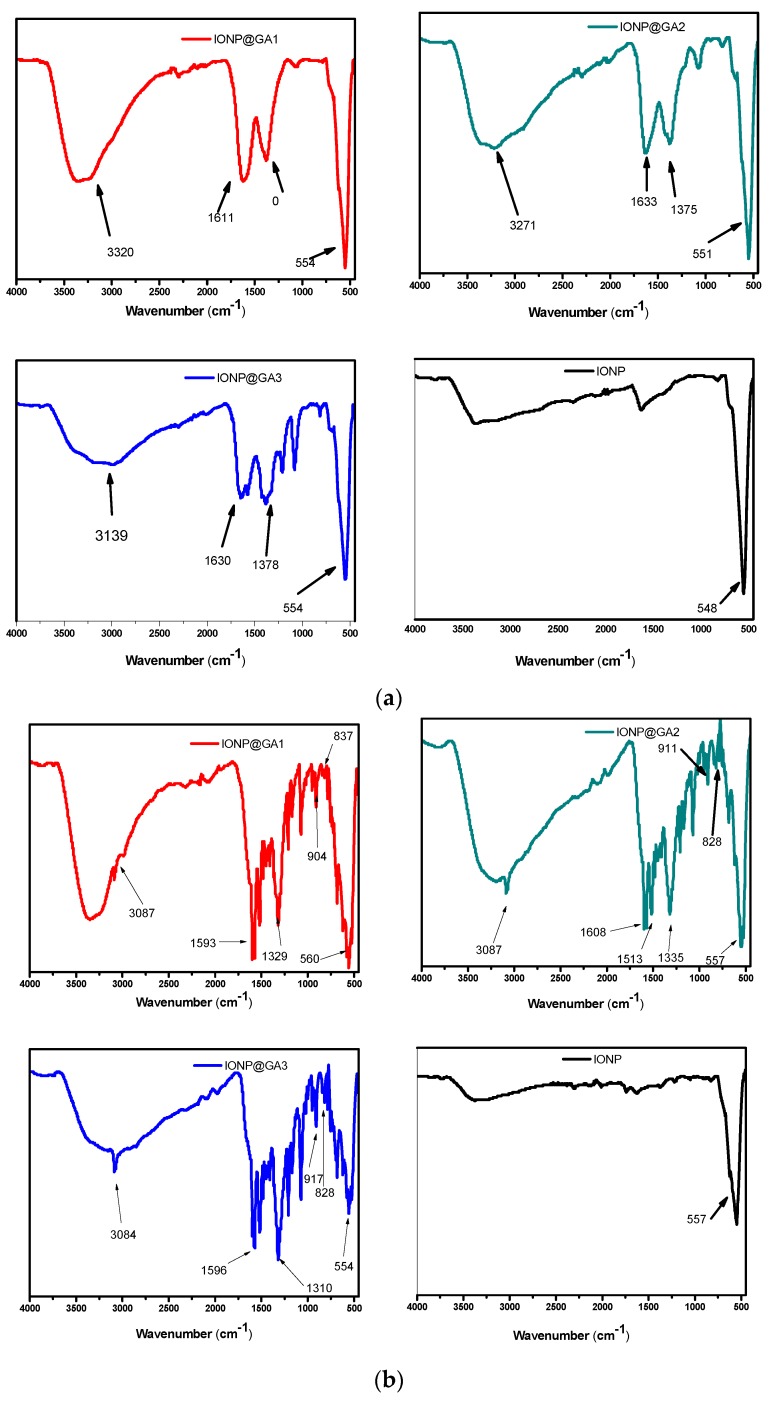
FTIR spectra of (**a**) IONP@GA before DPPH assay (**b**) IONP@GA after DPPH assay.

The FTIR spectra of Iron oxide nanoparticles (IONP), In situ functionalized iron oxide nanoparticles IONP@GA1, IONP@GA2 and post-functionalized IONP@GA3 are illustrated by Figure 1a. The peak at 550, 551, 554, and 554 cm^−1^ represent the characteristic Fe-O stretching of for IONP, IONP@GA1, IONP@GA2 and IONP@GA3, respectively [50]. Broad peak at 3100–3200 cm^−1^ refers to OH stretching of phenol. The peak at 1079, 1089, and 1078 cm^−1^ corresponds to Fe-O-C for sample IONP@GA1, IONP@GA2 and IONP@GA3, respectively. Peak at 1633, 1611, and 1630 cm^−1^ confirms the presence of carbonyl group in IONP@GA1, IONP@GA2, and IONP@GA3, respectively.

Further the surface reactivity was ascertained after 2,2-diphenyl-1-picrylhydrazyl (DPPH) assay. Figure 1b shows the FTIR spectra of IONP@GA samples after DPPH assay. IONP@GA samples were mixed with the excess DPPH. The mixture was kept in the dark for 30 min and then washed thrice with ethanol. N-O stretching gave two peaks at 1530, 1310, 1513, 1335, 1530, and 1329 cm^−1^ for IONP@GA3, IONP@GA1, and IONP@GA2, respectively. This confirms the attachment of DPPH radical (ESI, Figure S7) to the surface of IONP@GA. Not a single peak for DPPH radicals was appeared for IONP.

### 2.2. Raman Spectra

**Figure 2:**
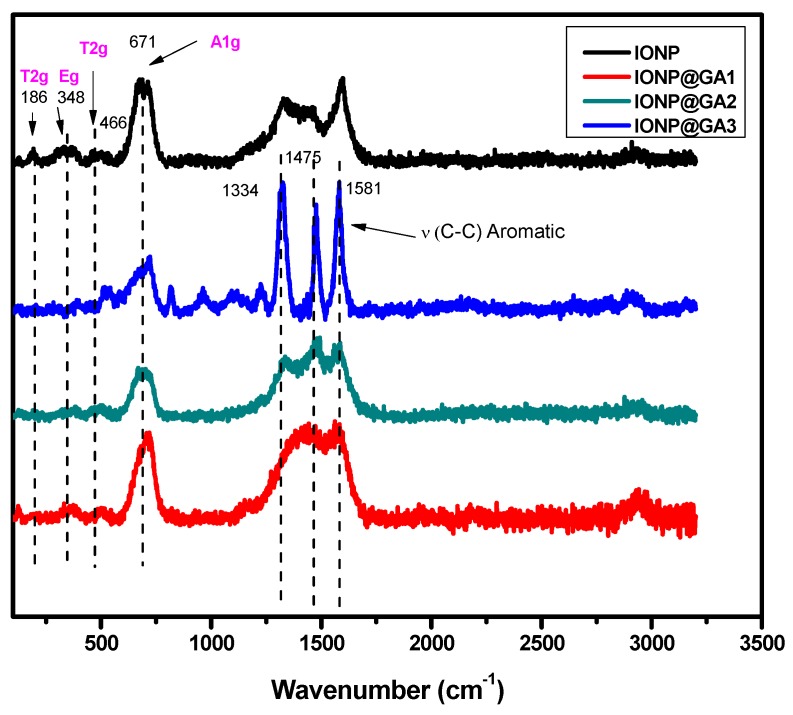
Raman spectra of IONP@GA.

Raman spectra of IONP@GA samples are shown in Figure 2. The structural phase of the synthesized nanoparticles is further supported by Raman spectroscopy that shows the band absorption at 671 cm^−1^ (A1g) is for magnetite [51]. In addition to main band absorption at 671 cm^−1^, all the samples show peaks at 466 cm^−1^ (T2g) and 348 cm^−1^ (Eg) of magnetite. Moreover, the Raman spectra also confirmed the absence of maghemite (ESI, Figure S8) [52,53].

### 2.3. X-ray Diffraction(XRD) Analysis

**Figure 3:**
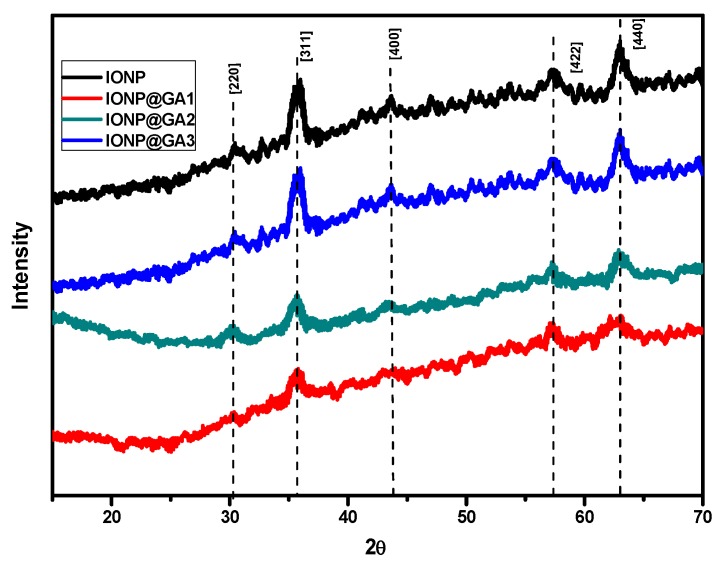
XRD spectra of unfunctionalized and functionalized IONP.

The XRD peak patterns of unfunctionalized and functionalized iron oxide nanoparticle are illustrated by Figure 3. XRD reflections shows pure magnetite nanoparticle with cubic inverse spinal structure in all the samples (JCPDS No. 82-1533) [22] ESI, Figure S9. Further, diffraction peaks for magnetite appeared at the 2*θ* value of 25°, 30°, 43°, 57°, and 63°, correspond to [311], [220], [400], [422] and [440] lattice planes, respectively. The absence of superlattice diffractions at [210], [213] and [300] confirms that maghemite is not present in any sample. Moreover, XRD data confirms that coating did not affect the phase of iron oxide.

### 2.4. Morphological Characterization

**Figure 4:**
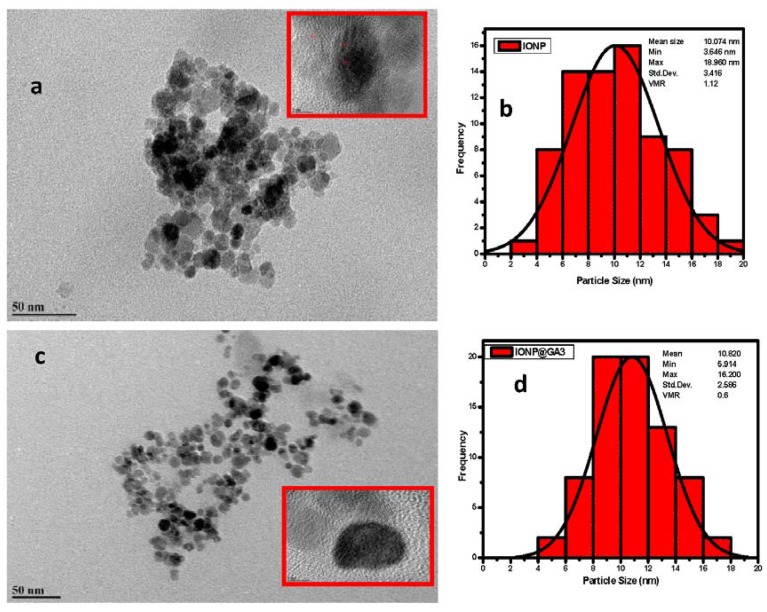

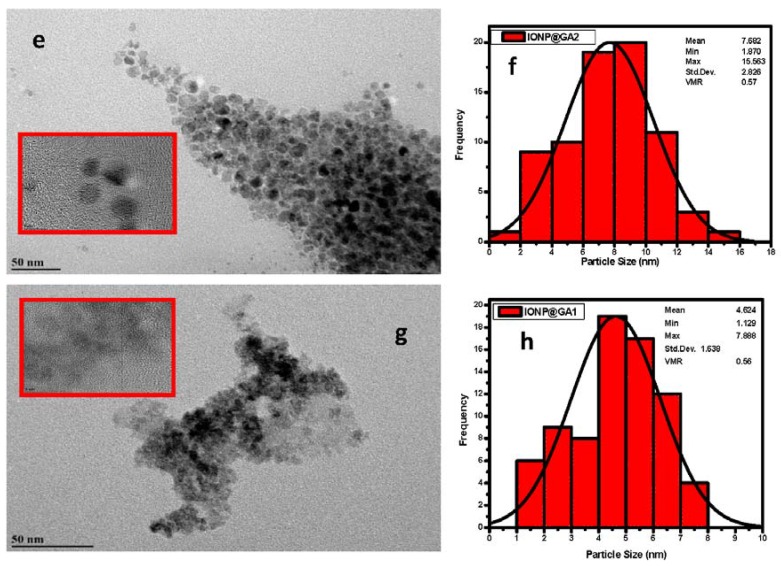
HRTEM images (**a**,**c**,**e**,**g**) and particle size distribution (**b**,**d**,**f**,**h**) of IONP@GA.

The morphology of the synthesized IONP@GA was analyzed using High Resolution Transmission Electron Microscopy (HRTEM). Figure 4 shows the HRTEM image with size distribution for GA functionalized magnetite nanoparticles. The average size for IONP@GA1, IONP@GA2, and IONP@GA3 and IONP are 5, 8, 10.8 and 10.0 nm, respectively (Figure 4b,d,f,h). It is clear from the image that the particles have spherical shape with uniform size distribution. Crystal lattice fringe spacing of 0.26 nm, corresponding to the [220] lattice planes in cubic iron oxide nanoparticles [54]. The agglomeration of nanoparticles occurs due to the magnetic behavior of the particles.

The in situ functionalized IONP@GA1 and IONP@GA2 have ultra-small particle size as compared to IONP and post functionalized IONP@GA3 as shown in Figure 4. This reveals that the in-situ functionalization process followed in this study has a strong and successful size-control effect, which is significantly lower than other synthesis routes.

**Figure 5:**
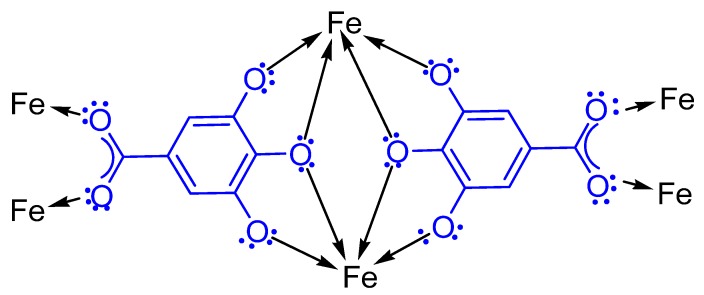
Proposed structure of iron gallate.

The remarkable size-control effect exerted by GA on in situ-functionalized IONP, can be attributed to iron cations chelate with GA (Figure 5) to form blue-black ferrous/ferric gallate [13,55]. In the same context, GA had minimized the IONP agglomeration, which might be due to either GA bonding site, which strongly coordinates with the IONP surface by forming a monolayer on the IONP surface, which leads to a decrease in magnetic dipole-dipole interaction among the aggregates during formation of nanoparticles and/or the presence of the bulky phenyl group in GA provides sufficient steric hindrance to minimize the IONP agglomeration. Moreover, GA has hydrophilic functional groups, which improves the solubility of IONP in polar solvents and could serve as potential H-bonding sites [56]. Overall, GA has proved an astounding ability to control particle size, solution stability, and hydrophilicity of the IONP nano-antioxidant system.

### 2.5. Magnetic Properties

**Figure 6:**
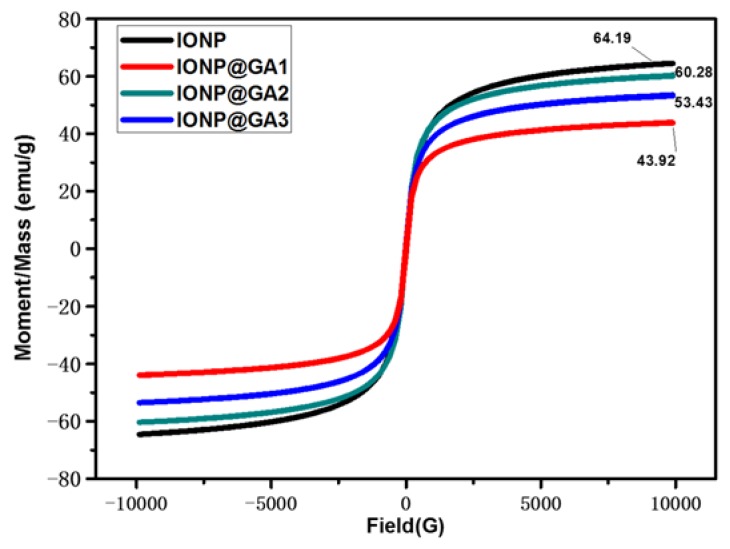
Magnetic hysteresis loops of IONP@GA.

Figure 6 shows the hysteresis loops as a function of the magnetic field at room temperature. The values of 64.19, 60.28, 53.43 and 43.92 emu g^−1^ were given for IONP, IONP@GA2, IONP@GA3, and IONP@GA1, respectively. The magnetic parameters, including saturation magnetization are shown in Table 1. The nanoparticles synthesized here are superparamagnetic with low magnetization values than the bulk magnetite (~92 emu g^−1^) [57]. Functionalized IONPs showed a decrease in saturation magnetization which was most likely due the decrease in saturation magnetization for functionalized IONPs was due to the presence of more organic contents and impurities on the surface of the magnetic nanoparticles [22,58,59].

### 2.6. Energy Dispersive X-ray Spectroscopy (EDX) Analysis

**Figure 7:**
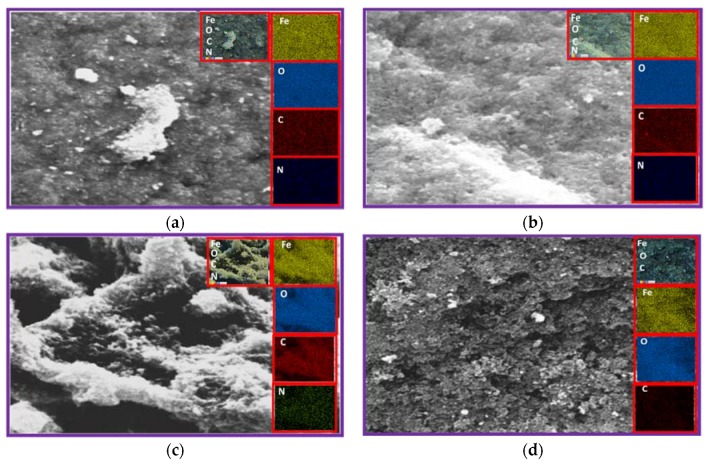
FESEM image (**a**) IONP@GA1 (inset: EDX elemental map of Fe, O, C, and N); (**b**) IONP@GA2 (inset: EDX elemental map of Fe, O, C, and N); (**c**) IONP@GA3 (inset: EDX elemental map of Fe, O, C, and N); and (**d**) IONP (inset: EDX elemental map of Fe, O, C).

Fe and O signals confirmed the presence of magnetite nanoparticles, while the C signals are derived from the organic matrix as shown in Table 2 (ESI, Figures S1–S4). N signals were also observed in sample IONP@GA1 and IONP@GA2 which might be due to formation of ferrous/ferric gallate during synthesis. The spatial distribution of the iron atoms observed from the mapping images, clearly indicates the uniform distribution of the atoms. Figure 7a–d represents the EDX of IONP@GA1, IONP@GA2, IONP@GA3, and IONP, respectively, after conducting DPPH assay.

The EDX mapping after the DPPH assay, indicates an increase in the percentage of carbon and nitrogen, which could be strongly attributed to attachment of DPPH radicals on the surface of IONP@GA to form IONP@GA-DPPH, which is in agreement with the FTIR results. Nitrogen contents increased from 0% to 0.8%, 0.4% to 0.6%, and 0.6% to 0.3% for IONP@GA1, IONP@GA2, and IONP@GA3 respectively, while carbon contents increased from 7% to 7.2%, 4.8% to 6%, and 10.8% to 11.7% for IONP@GA1, IONP@GA2, and IONP@GA3, respectively. No change in nitrogen contents for unfunctionalized IONP was observed, which indicated that free radicals could not attach to the IONP surface.

### 2.7. Prediction Activity Spectra of Substances (PASS) of Biological Activity

PASS predictions have been applied to design of new potent free radical inhibitors in phenol series as potential antioxidant drugs [4,60,61]. PASS provides simultaneous predictions over 4000 kinds of biological activity with mean accuracy of 95% [62,63,64,65]. The outcome of prediction is available Table 3 as list of activities with appropriate Pa (probability “to be active”) to Pi (probability “to be inactive”) ratio for GA. It is reasonable that only those types of activities may be revealed by the compound, which Pa > Pi [66]. A portion of the predicted biological activity spectra for the GA is given in Table 3.

### 2.8. Antioxidant Activity

**Figure 8:**
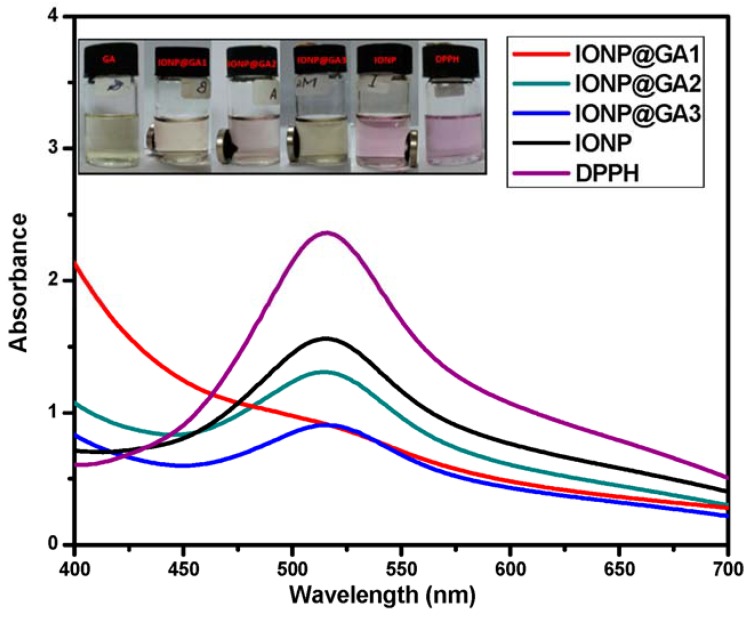
UV-VIS spectra.

The color of the DPPH solution in the presence of the functionalized iron oxide changes gradually from deep violet to pale yellow, which provides the visual monitoring of the antioxidant activity of the nanoparticles. From the UV-VIS absorption curve in Figure 8, it can be inferred that the peak intensity of DPPH is lowering. The free radical scavenging percentage is calculated from the decrease in absorbance at 517 nm. In the DPPH scavenging assay, the IC50 value (Table 4) and the inhibition of stable DPPH free radicals of the compounds were found to be IONP@GA1 (2.7 ± 0.003 mg/mL; 61%), IONP@GA2 (2.2 ± 0.002 mg/mL; 59%) and IONP@GA3 (IC50 1 ± 0.003 mg/mL; 78%) at 10^−4^ M, which are 2–4 fold more than the unfunctionalized IONP (IC50 4.7 ± 0.002 mg/mL; 50%) (Table 4) as a reference in this assay. The DPPH scavenging of four samples was found to be in the order of IONP@GA3 > IONP@GA1 > IONP@GA2 > IONP. The free radical scavenging is most probably due to electron transfer from IONP@GA to free radicals located at the central nitrogen atom of DPPH. Enhanced free radical scavenging for IONP@GA is due to the synergistic effect of IONP and GA.

### 2.9. Antimicrobial Activity

#### 2.9.1. Antibacterial Activity

**Figure 9:**
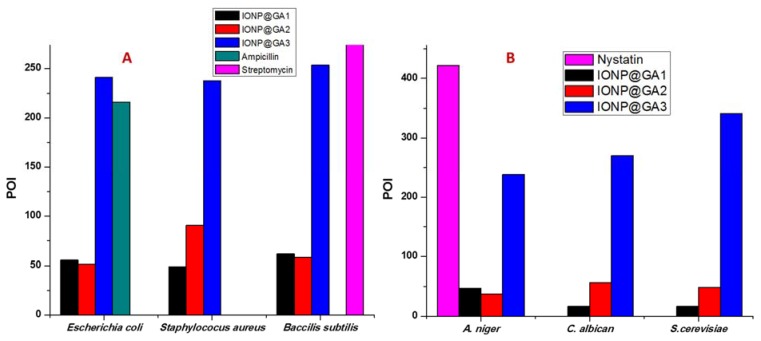
Percentage of inhibition (POI) of (**A**) bacterial growth and (**B**) mycelia growth of fungi, after treatment with IONP@GA.

Figure 9A shows the results of agar well diffusion test expressed as percentage inhibition of diameter growth (PIDG) of IONP at a concentration of 100 mg/mL. All three types of functionalized IONP@GA showed antibacterial activity on both Gram-positive and -negative strains. However, the highest growth inhibition percentages were observed upon using IONP@GA3, in which all bacterial strains scored high inhibition values. Thus, revealing its prominent and powerful bactericidal effect.

Generally, results showed a variable IONP@GA antibacterial activity among different bacterial strains. Such trend can be explained based on cell wall composition of each type. Upon administration of IONP@GA, bacterial growth inhibition is believed to occur through the penetration of functionalized IONPs into cells with subsequent cell wall damage by breaking *β*-1,4-glyosidic bond. Nevertheless, nano-antioxidant IONP@GA compounds were proven to exhibit antibacterial activity on both bacterial strains.

#### 2.9.2. Antifungal Activity

The antifungal activity of IONP@GA against *Aspergillus niger*, *Candida albicans*, and *Saccharomyces cerevisiae* was investigated using the well diffusion method. Results, as presented in Figure 9B, show a potent antifungal activity of tested compounds among all fungi strains used. The highest percentage of inhibition (POI) was obtained by utilizing IONP@GA3 compound. Together with its antibacterial activity, these results confirm the higher antimicrobial activity of IONP@GA3 compared to other functionalized IONP compounds. The mechanism responsible for antifungal activity seen, can be assumed to involve the attachment of nanoparticles to the respiratory sequence, which leads to cell death [67].

**Figure 10:**
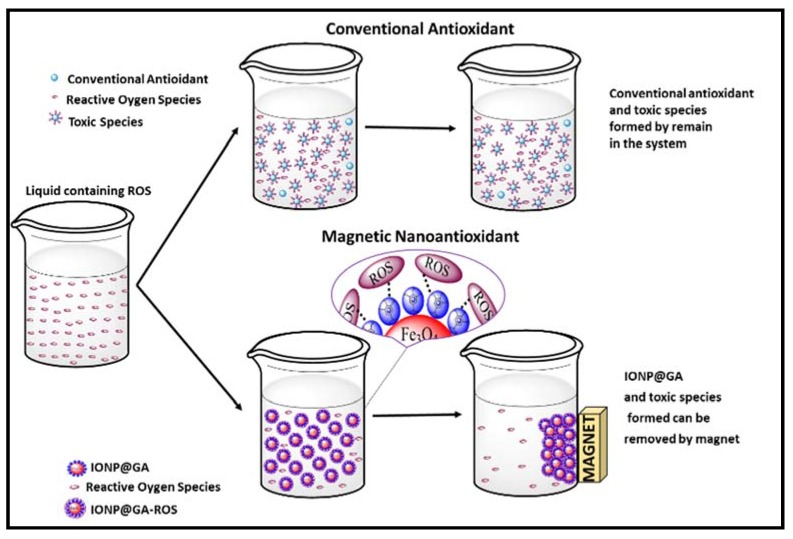
Proposed potential applications of IONP@GA in industry.

With reference to the above discussed results, the synthesized nano-antioxidant IONP@GA in this study had proven to own strong antimicrobial properties, that potentiate various biomedical applications. Yet, the astonishing antioxidant activity of IONP@GA compounds may find further applications in industrial fields. Herein, we propose a potential application of nanomagnetite in industries as illustrated in Figure 10. We propose that controlling the size and antioxidant activity is a promising method for improving industrial based materials. These magnetic nanoantioxidants have many advantages over conventional antioxidants. In addition to the GA functional moiety that enables less toxic, biocompatible coating with fine size-control, the mixture of IONP@GA and toxic materials (IONP@GA-ROS) formed during the reaction of nanomagnetite with ROS can be easily removed from the system by a magnet and can be recycled.

## 3. Discussion

GA functionalized organic nanomagnetites have been successfully synthesized via in situ and post-functionalization techniques. The average particle size is 5 and 8 nm for in situ-functionalized nanomagnetie IONP@GA1 and IONP@GA2, respectively, while it is 11 nm for post-functionalized IONP@GA3. The particle size fall in order of IONP@GA3 > IONP > IONP@GA2 > IONP@GA1. Addition of GA during in-situ synthesis had controlled the particle size of IONP, thus efficiently overcoming a critical obstacle in nano-antioxidant synthesis. PASS-predicted antioxidant values and other biological activities for the GA indicated that the GA-functionalized IONP surface could be likely to reveal these activities, in addition to the iron oxide activities and its biocompatibility as multi-purpose tools for guided drug delivery and bioimaging. The DPPH scavenging of the four samples is found to be in the order of IONP@GA3 > IONP@GA1 > IONP@GA2 > IONP. The in situ and post functionalization methods successfully improved the free radical scavenging of IONP to be more than 2–4 fold. The present investigation highlights the synergistic effect of the magnetite and GA, which leads to the enhancement of the free radical scavenging capacity of IONP@GA. The synthesized IONPs@GA samples are hydrophilic and exhibited greater antioxidant activity and degraded the DPPH radicals efficiently. FTIR and EDX techniques confirmed that IONP@GA is scavenged the DPPH radicals and yielding IONP@GA-DPPH composite suggesting that free radicals can be easily removed from the system by magnet and can be recycled. Finally, IONP@GA showed potential antimicrobial activity (antibacterial and antifungal) effects on strains tested, probably through the destruction of membrane integrity; therefore, it was concluded that IONP@GA has considerable antimicrobial activity, which is promising for further clinical applications. The methodology used here can be employed for synthesizing organic nanocompounds with other antioxidants that are magnetically separable, efficient, low cost and may have potential applications in polymer, cosmetics, biomedical and food industry.

## 4. Materials and Methods

### 4.1. Materials

Ferrous chloride tetrahydrate (FeCl_2_·4H_2_O, Merck, Mendota Heights, MN, USA), ferric chloride hexahydrate (FeCl_3_·6H_2_O, Sigma ≥ 97%, (Saint Louis, MO, USA)), Gallic acid (R and M) and ammonia solution (R and M, 28%) (Shanghai, China) were used as received without further purification. Analytical grade reagents were used during all experiments. The shape and size of samples were analyzed using a (JEOL JEM-2100F, Tokyo, Japan). Phase identification and crystallinity of the magnetite samples were determined using a PANalytical X-ray diffractometer (model EMPYREAN, Almelo, The Netherlands) where Cu-Kα (λ = 1.54060 Å) radiation was used. The spectra has been recorded for 2θ ranging from 10.00 to 90.00. FTIR of the samples was recorded using a Spectrum 400 (PerkinElmer, Boston, MA, USA). EDX was studied using EDX (INCA Energy 200 (Oxford Inst.), Hillsboro, OR, USA). The percentage composition was determined using the surface area method. The Raman spectra were obtained from Renishaw inVia Raman (Gloucestershire, UK) by using a 514 nm argon gas laser. The magnetism hysteresis loop was obtained from a Lake Shore vibrational sample magnetometer (VSM) (Westerville, OH, USA) at room temperature in a magnetic field range of −10 to +10 kOe.

### 4.2. Synthesis of IONP

Ferrous chloride and ferric chloride with a molar ratio of 1:1.5 was dissolved in 100 mL deionized water. Ammonium hydroxide solution (3.0 M) was added 5.0 mL min^−1^ into the solution at a stirring speed of 600 rpm to reach a final pH 11. The reaction was carried out at 80 °C under an oxidizing environment and stirring was continued for another 90 min. The resultant black precipitate was isolated by magnetic decantation. The precipitate was rinsed with deionized water and ethanol. After that, it was freeze dried.

#### 4.2.1. In Situ Functionalization

##### Synthesis of Organic IONP@GA1

Ferrous chloride and ferric chloride with a molar ratio of 1:1.5 was dissolved into 100 mL deionized water, followed by the addition of 1 g GA. 3.0 M of ammonium hydroxide solution was added 5.0 mL min^−1^ into the solution at 600 rpm stirring speed to reach a final pH 11. The reaction was conducted at 80 °C under oxidizing environment with continued stirring for another 90 min. The resultant black precipitate was isolated by magnetic decantation. Lastly, the precipitate was rinsed with deionized water and ethanol, then freeze-dried.

##### Synthesis of Organic IONP@GA2

Ferrous chloride and ferric chloride with a molar ratio of 1:1.5 was dissolved in 100 mL deionized water. Ammonium hydroxide solution (3.0 M) was added at a speed of 5.0 mL min^−1^ into the solution at a stirring speed of 600 rpm to reach a final volume of 100 mL. One gram of GA was added to the reaction mixture. The reaction was carried out at 80 °C under an oxidizing environment with continued stirring for another 90 min. The resultant black precipitate was isolated by magnetic decantation. Finally, the precipitate was rinsed with deionized water and ethanol, then freeze-dried.

#### 4.2.2. Post-Functionalization

##### Synthesis of IONP@GA3

One gram of GA was mixed with the IONP and was kept under stirring for 24 h. The precipitate was rinsed with deionized water and ethanol, then freeze-dried.

#### 4.2.3. Antioxidant Activity

Various chemical based assays have been used to determine antioxidant activities. Based on the reaction involved, these assays can be classified into two main types. Hydrogen atom transfer and electron transfer. In this present work, the DPPH assay involving electron transfer was selected to study the antioxidant activity of functionalized magnetite nanoparticles [45]. The antioxidant activity was determined by using a standard DPPH method with some modification [44,45]. Sample stock suspensions in methanol (300 μL) and 1 mL of methanolic solution of DPPH (0.2 mM) were mixed in a 1 cm quartz cuvettes. Absorbance measurements were taken after 30 min. The decrease in absorbance at 517 nm was observed continuously. All the experiments were duplicated. All the measurements were taken within exactly 30 min after mixing the sample with DPPH solution. The radical scavenging activity was calculated using Equation (1):

(1) $$\text{Percentage}\;\text{of}\;\text{Inhibition}\;(\%)=\frac{\left(Ac-As\right)}{Ac}\times 100$$

In which *As* = the absorbance of the compounds/positive control and *Ac* = the absorbance of the control (DPPH solution). To determine the concentration required to achieve 50% inhibition (IC_50_) of the DPPH radical; the percentage of DPPH inhibition for each compound was plotted against different concentrations.

#### 4.2.4. Antimicrobial Activity

##### Determination of Antibacterial Activity

The antibacterial activities of the IONP@GA were estimated using the agar well diffusion method [68]. Precultures of *Staphylococcus aureus*, *Bacillus substilis*, and *Escherichia coli* were spread on the surface of nutrient agar (NA) agar and wells (diameter = 6 mm) were filled with 100 μL of the test samples (100 mg/mL) and incubated at 37 °C for 24 h. Sterile distilled water was used as a negative control. Positive controls used were streptomycin 100 mg/disc and ampicillin 100 mg/mL for Gram-positive and Gram-negative bacteria, respectively. The formation of the halo (inhibition) zone and the diameter of inhibition zones were determined to evaluate the antibacterial properties.

##### Determination of Antifungal Properties

For antifungal properties, all IONP@GA types were tested against *Aspergillus niger*, a filamentous fungus (multicellular); *Saccharomyces cerevisiae*, a yeast (unicellular); and *Candida albican*, a yeast using the well diffusion method. PDA agar plates were inoculated with fungal strains under aseptic conditions and wells (diameter = 6 mm) were filled with 100 μL of the test samples (100 mg/mL) and incubated at 25 °C for 48 h. Sterile distilled water was used as negative control. The positive control used was nystatin at 100 mg/mL. The percentage of inhibition (POI) of mycelia growth was calculated using Equation (2):

(2) $$POI=\frac{R1-R2}{R1}\times 100$$

where

*R*1 = radius of the pathogen away from the antagonist.
*R*2 = radius of the pathogen towards the antagonist.

## Figures and Tables

**Table 1:** Saturation magnetizations of IONPs.

Sample	*Ms* (emu g^−1^)
IONP	64.19
IONP@GA1	43.90
IONP@GA2	60.26
IONP@GA3	53.43

**Table 2:** EDX elemental composition (**A**) Before DPPH assay (**B**) After DPPH assay.

Sample	A				B			
	Fe	O	C	N	Fe	O	C	N
IONP	69.6	39.4	-	-	77.5	21.2	1.3	-
IONP@GA1	62	30.2	7	0.6	62.6	29.5	7.2	0.8
IONP@GA2	65.6	29.1	4.8	0.4	63.7	29.7	6	0.6
IONP@GA3	58.7	30	10.8	-	61.5	26.4	11.7	0.3

**Table 3:** Predicted biological activity spectra of the GA on the basis of PASS prediction software.

Biological Activity	Pa ^a^	Pi ^b^
Antioxidant	0.529	0.005
Free Radical Scavenger	0.579	0.007
Lipid Peroxidase Inhibitor	0.554	0.012
Anti-inflammatory	0.560	0.041
Antibacterial	0.420	0.026
Antibacterial, ophthalmic	0.255	0.005
Antifungal	0.255	0.050
Antifungal (Pneumocystis)	0.109	0.003

^a^ Probability “to be active”; ^b^ Probability “to be inactive”.

**Table 4:** IC50 of IONP@GA.

IC50 ^a^ Values (mg/mL) ± S.E.M ^b^ and Max. Inhibition %			
Sample		IC50 mg/mL	% Inhibition
IONP@GA3	5 mg	1.00 ± 0.003	78
IONP@GA2	5 mg	2.2 ± 0.002	59
IONP@GA1	5 mg	2.7 ± 0.003	61
IONP	5 mg	4.7 ± 0.002	50

^a^ IC50: 50% effective concentration; ^b^ S.E.M: standard error of the mean.

## Supplementary Materials

The following are available online at http://www.mdpi.com/2079-4991/7/10/306/s1.
