# Supplementary material for: Surface Functionalization of Iron Oxide Nanoparticles with Gallic Acid as Potential Antioxidant and Antimicrobial Agents
Source: Nanomaterials (Basel). 2017 Oct 5;7(10):306. doi: 10.3390/nano7100306 (PMC5666471; doi:10.3390/nano7100306)
Supplement: Supplementary file 1 [file nanomaterials-07-00306-s001.pdf]

Supporting Information

Surface Functionalization of Iron oxide Nanoparticles with Gallic Acid as Potential Antioxidant and Antimicrobial agents

Syed Tawab Shah [1], Wageeh A. Yehye [1,*], Omar Saad [2], Khanom Simarani [3], Zaira Zaman Chowdhury [1,*], Abeer A. Alhadi [2] and Lina A. Al-Ani [1]

[1] Nanotechnology & Catalysis Research Centre (NANOCAT), University of Malaya, Block A, Level 3, Institute of Postgraduate Studies Building, Kuala Lumpur 50603, Malaysia; tawab_shah2003@yahoo.com (S.T.S.); linaalani@ymail.com (L.A.A.-A.)

[2] Department of Pharmacy, Faculty of Medicine, University of Malaya, Kuala Lumpur 50603, Malaysia; omar79@siswa.um.edu.my (O.S.), abeer@um.edu.my (A.A.A.)

[3] Institute of Biological Sciences, Faculty of Science, University of Malaya, Kuala Lumpur 50603, Malaysia; hanom_ss@um.edu.my (K.S.)

* Correspondence: wdabdoub@um.edu.my (W.A.Y.); dr.zaira.chowdhury@um.edu.my (Z.Z.C.); Tel.: +60-3-7967-2924 (W.A.Y.); +60-3-7967-6954 (Z.Z.C.); Fax: +60-3-7967-6956 (W.A.Y.); +60-3-7967-6556 (Z.Z.C.)

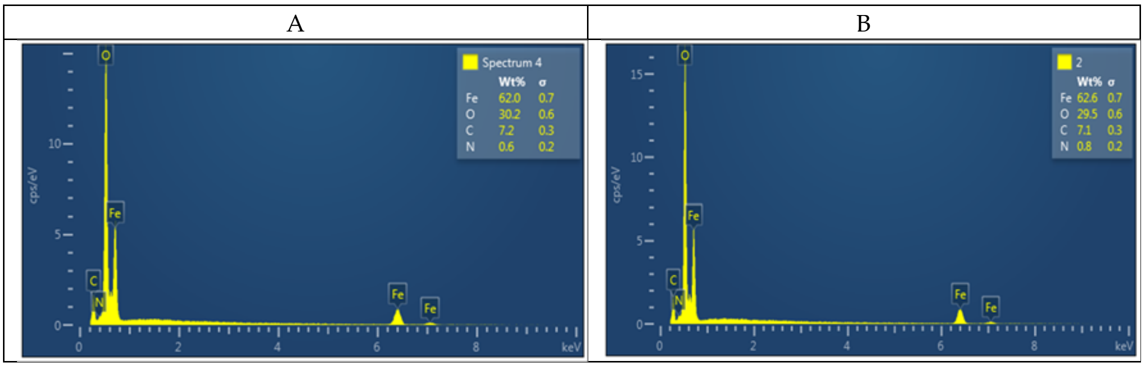

Figure S1 EDX spectrum of IONP@GA1 **A**) Before DPPH assay **B**) After DPPH assay.

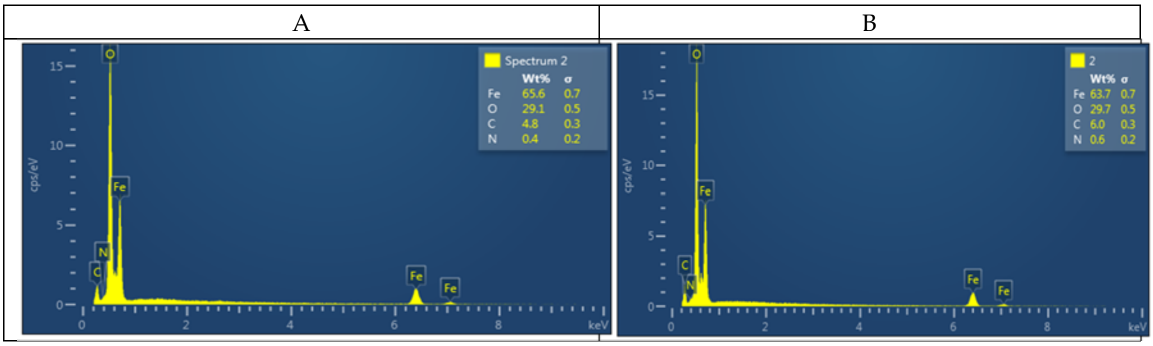

Figure S2 EDX spectrum of IONP@GA2 A) Before DPPH assay B) After DPPH assay.

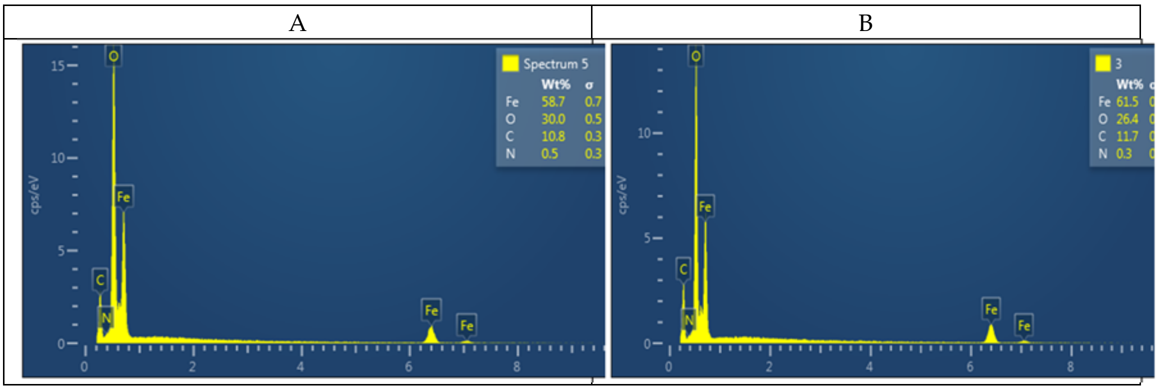

Figure S3 EDX spectrum of IONP@GA3 A) Before DPPH assay B) After DPPH assay.

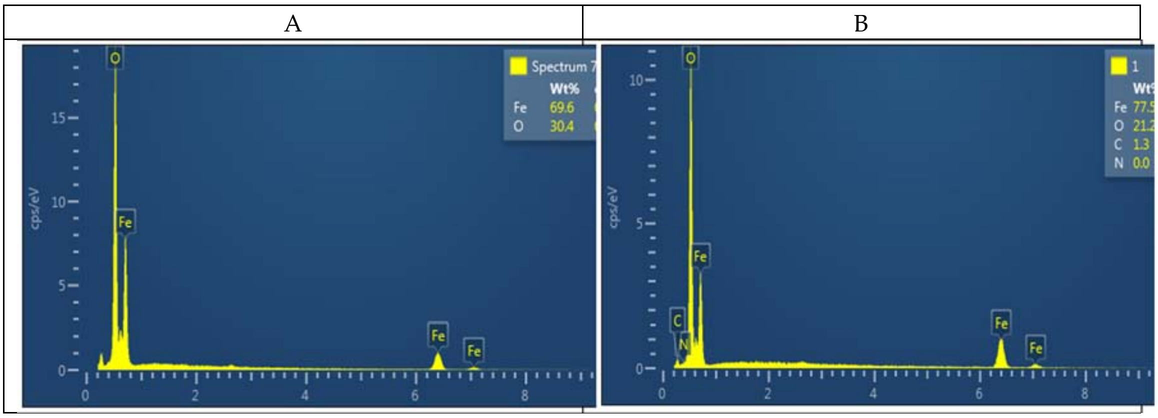

Figure S4 EDX spectrum of IONP A) Before DPPH assay B) After DPPH assay.

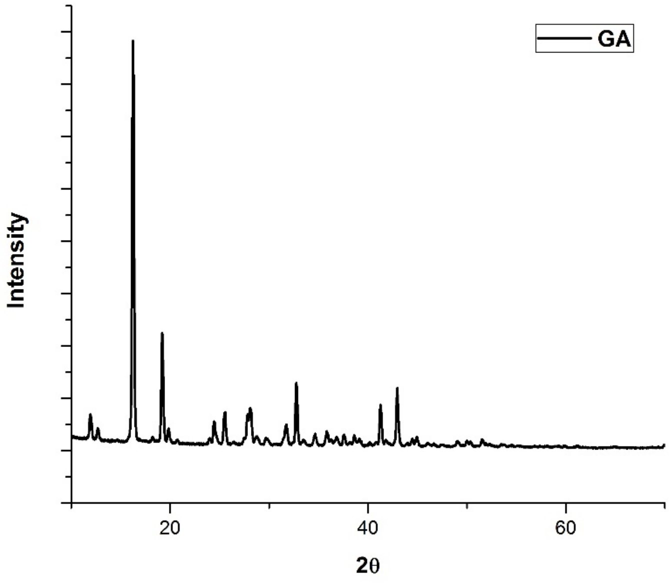

Figure S5. XRD Spectra of gallic acid.

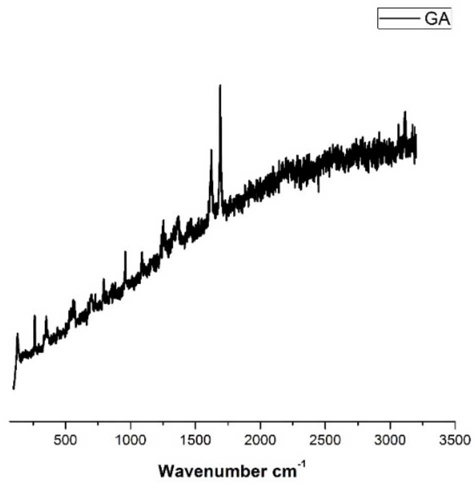

Figure S6. Raman Spectra of gallic acid.

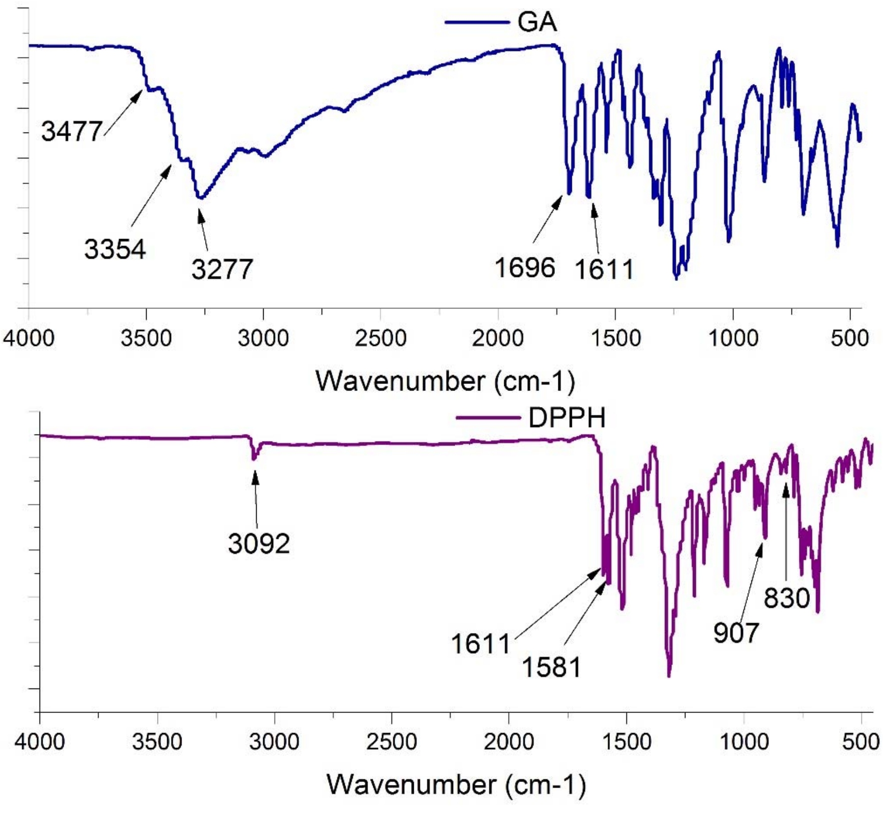

Figure S7. FTIR specta of gallic acid and DPPH.

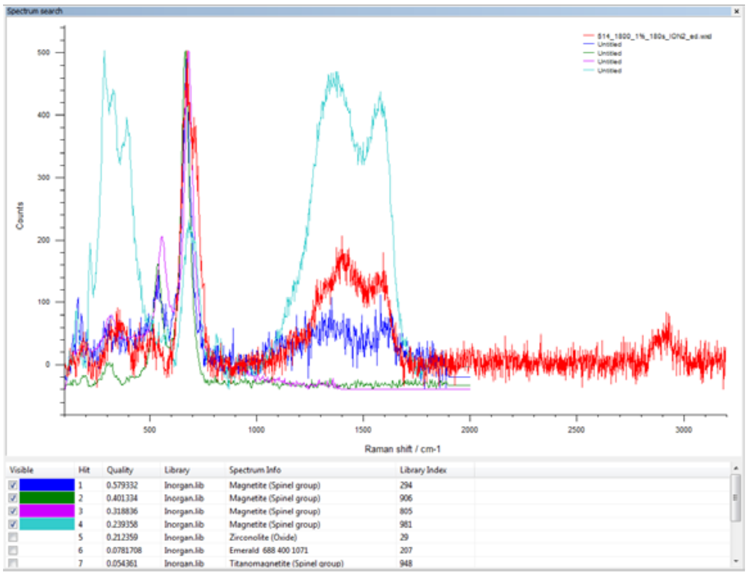

Figure S8. Comparison of Raman Spectra of IONP with commercial Magnetite.

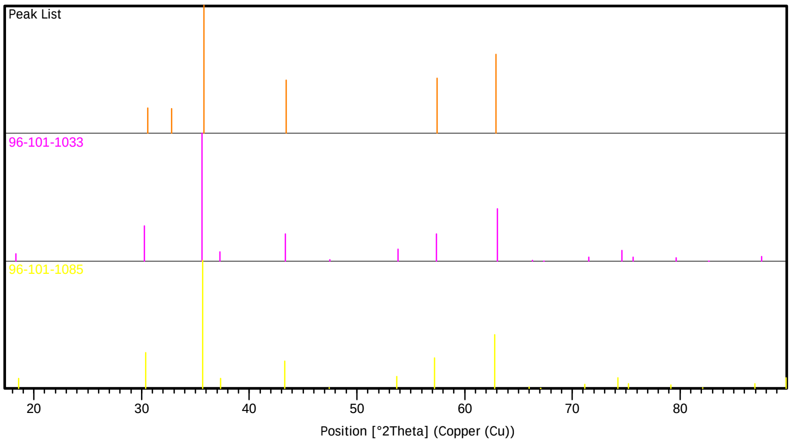

Figure S9. XRD pattern of IONP@GA and JCPDS No 96-101-1033.

Table S1. IC50 of IONP@GA

Conc. (mg)	IONP@GA1	IONP@GA2	IONP@GA3	IONP
5	61	59	78	50
2.5	53	53	70	44
1.25	43	46	47	42
0.625	33	43	46	34
